# P. M. B. Association of Texas

**Published:** 1888-08

**Authors:** 


### P. M. B. ASSOCIATION OF TEXAS.

The receipt of Mrs. Glover, published below, should have been received in time for the last issue, but it was delayed in consequence of the Secretary sending the money to Eagle Lake, instead of Eagle Springs.

| | |
|---|---|
| The total amount collected from 126 members was . . . | $126 00 |
| Expenses of collecting, to-wit: 147 postal cards, printing same, postage in return cards, exchange on draft and P. O. M. order . . . . . . . . . . . . . . . . . . . | 5 00 |
| Net amount sent Mrs. Glover . . . . . . . . . . . . . | $121 00 |

Received of Drs. W. A. Morris, President, and F. E. Daniel, Secretary and Treasurer P. M. B. A. of Texas, one hundred and twenty-one dollars, collected for Dr. Glover's certificate.

MRS. WM. GLOVER.

EAGLE SPRINGS, Texas, August 10, 1888.

Of the twenty-one members who have not paid this assessment (No. 6) there may be some who will yet pay, and if so, the amount collected will be forwarded.

Apropos to the subject the following letter is published, with consent of writer. [In reply we will say, that next month, September, we will publish in the JOURNAL, and send out in circular form, AN ADDRESS TO THE PROFESSION, from the Board of Directors, which, we hope, will have the desired effect; and as an additional inducement we are offering membership free, for thirty days, as premium for subscribers to the JOURNAL. See notice.]

MIDLOTHIAN, Texas, August 18, 1888.

DEAR DR. DANIEL—Inclosed please find one dollar P. M. order on assessment No. 7. The report of the Association is so discouraging that it is with a degree of reluctance that I respond. I have been a member now about two years, and it numbers less, I believe, than when I became a member. It does seem to me, unless there is an effort made to arouse the profession in Texas to a responsive sense of support in this grand and noble association, we had better give up the enterprise. Hoping for a better report next time, I am fraternally yours,

Z. T. BUNDY.

